# Protocol and Evaluation of 3D-Planned Microsurgical and Dental Implant Reconstruction of Maxillary Cleft Critical Size Defects in Adolescents and Young Adults

**DOI:** 10.3390/jcm10112267

**Published:** 2021-05-24

**Authors:** Krzysztof Dowgierd, Rafał Pokrowiecki, Maciej Borowiec, Zuzanna Sokolowska, Martyna Dowgierd, Jan Wos, Marcin Kozakiewicz, Łukasz Krakowczyk

**Affiliations:** 1 Head and Neck Surgery Clinic for Children and Young Adults, Department of Clinical Pediatrics, University of Warmia and Mazury, ul. Oczapowskiego 2, 10-719 Olsztyn, Poland; krzysztofdowgierd@gmail.com; 2 Head and Neck Surgery Department—Maxillofacial Surgery Department, Craniofacial Center, Regional Specialized Children’s Hospital, ul. Zolnierska 18A, 10-561 Olsztyn, Poland; borowiecmaciej@gmail.com (M.B.); sokolowskazuzanna@gmail.com (Z.S.); 3 Center of Craniofacial Malformations for Children and Young Adults, Regional Specialized Children’s Hospital, ul. Zolnierska 18A, 10-561 Olsztyn, Poland; rejestracjatwarzoczaszki@wssd.olsztyn.pl; 4 Department of Laryngology, Stefan Zeromski Hospital, Os. Na Skarpie 66, 31-913 Cracow, Poland; mjwos@cmwip.pl; 5 Department of Maxillofacial Surgery, Medical University in Lodz, Pl. Hallera 1, 90-647 Łodz, Poland; marcin.kozakiewicz@umed.lodz.pl; 6 Branch in Gliwice, Oncological and Reconstructive Surgery Clinic, Maria Sklodowska-Curie Institute—Oncology Centre (MSCI), ul. Wybrzeze Armii Krajowej 15, 44-100 Gliwice, Poland; lukaszkrakowczyk@wp.pl

**Keywords:** cleft lip and palate, maxillofacial surgery, microsurgery, reconstruction, CMF, 3D planning, biomaterials, plastic surgery

## Abstract

Functional and esthetic final reconstruction of the cleft maxilla is still challenging. Current reconstructive and augmentation techniques do not provide sufficient bone and soft tissue support for the predictable rehabilitation with dental implants due to presence of maxillary bone critical size defects and soft tissue deficiency, scaring and poor vascularity. In this article the protocol for the use of 3D virtual surgical planning and microvascular tissue transfers for the reconstruction and rehabilitation of cleft maxilla is presented. Twenty-five patients (8 male/17 female) aged 14–41 years old with cleft-associated critical size defects were treated by 3D-virtual planned microvascular tissue transfers taken either from fibula, iliac crest, radial forearm, or medial femoral condyle. Follow-up lasted 1–5 years. No significant bone resorption (*p* > 0.005) nor volume loss of the graft was observed (*p* = 0.645). Patients received final permanent prosthetic reconstruction of the anterior maxilla based on 2–5 dental implants, depending on the defect severity. This is the first study presenting the use of virtual planning in the final restoration of the cleft maxilla with microvascular tissue transfers and dental implants. Presented protocol provide highly functional and aesthetic results.

## 1. Introduction

Cleft lip and palate (CLP) is a birth defect that disturbs the continuity of the upper lip, gingiva, alveolus, and palate tissues. The aim of primary palatoplasty and cleft lip reconstruction, which are performed in the early months/years of the child’s life, is to restore proper function to these orofacial structures, critical for food intake, breathing and speech development. The major objectives of the primary closure of the cleft are to provide anatomical closure of the defect, to create an apparatus for the development of normal speech and to minimize maxillary growth disturbances and dento-alveolar deformities [1]. However, regardless of the operative technique and positive outcomes, primary surgery always results in the development of tissue scarring and subsequent deformation of the lip and alveolus, which are additionally enhanced by the child’s growth. Maxillary deficiency and scarring results in sagittal, vertical, and horizontal hypoplasia, leading to the development of anterior and/or posterior crossbite during early dentition [2].

Secondary surgery procedures, along with early orthodontic and dental treatments, focus on preserving and restoring the alveolar bone. Secondary corticocancellous bone grafting is considered in order to improve maxillary growth and to provide alveolar continuity for the eruption and better alignment of the permanent canines and lateral incisors. Grafting is also performed to stabilize the premaxilla segment and support the nasal base. Traditionally, the procedure is performed in 9–11 year-old patients, but some authors suggest that earlier attempts (<6 year-old patients) are more beneficial for subsequent orofacial growth [3,4].

However, in individuals with wide clefts there is still a significant risk of secondary deformations, loss of premaxilla and permanent dentition, disruption of the previously reconstructed alveolus, fistula formation, bone graft resorption, and tissue scarring.

Alveolar deformations and maxilla discontinuities impede food intake and proper oral hygiene, contribute to the development of speech problem, and lead to the need for reconstruction with prosthetic devices. The amount of the residual bone graft remaining from previous reconstructions is insufficient to place dental implants. Re-augmentation by additional free bone grafts is insufficient at this stage of alveolar deformation due to the impossibility of stable fixing and stabilizing of the graft, progressive tissue scarring, poor vascularity and the limited regeneration potential of the cleft tissues [5,6,7]. Local flap reconstruction by means of buccal, tongue, temporal nasolabial, or temporal flaps is insufficient, as each is limited only to soft tissue reconstruction. It may be beneficial in the closure of wide naso-oral fistulas, but it does not resolve the problem of a hard tissue deficiency [8,9,10]. Severe deformations are usually regarded as critical, and hence require more complex reconstruction of both the hard and soft tissues in order to provide sufficient and functional reconstruction of the cleft maxilla [6,11]. Critical defects can be treated either by maxillary advancement, Le-Fort I osteotomy and distraction osteogenesis or microvascular tissue transfer [12,13,14] Maxilla in cleft patients is often difficult to mobilize due to scarring from previous procedures, and such patients have a greater affinity to relapse than non-cleft patients [11]. Moreover, despite being regarded as simple and cost-effective, distraction osteogenesis has major flaws. Cases of undesirable movement, changes in inclination of the teeth in the transport segment and increased tooth tipping, which require subsequent orthodontic retraction, have been reported. Incomplete closure of the bony defect on the nasal side of the alveolar cleft and the necessity for bone grafting anyway have also been described [15]. When a cleft maxilla with critical size defect (CSD) requires multilevel reconstruction at the soft and hard tissue levels, microvascular tissue transfer may be indicated [13]. The aim of such reconstruction is the comprehensive three-dimensional reconstruction of the alveolar bone, closure of fistulas, augmentation of the soft tissues, and stabilization of the premaxilla and/or maxilla bones for subsequent rehabilitation with dental implants or corrective jaw surgery.

Microvascular tissue transfers require meticulous pre-operative planning; therefore, 3D virtual surgical planning (3D-VSP) is of great importance for reconstructive surgery of the head and neck. 3D-VSP has garnered much interest recently and has clinically proven applicability in the reconstruction of post-resection defects of the mandible [16].

## 2. Materials and Methods

The ethics approval for this study was obtained from the Maria Skłodowska-Curie Memorial Cancer Center Ethics Committee in Gliwice (KB 430-15/17). Patients were diagnosed and operated at Regional Specialized Children’s Hospital, Olsztyn, Poland.

Patients and their legal guardians provided informed consent. A total of 25 patients (8 male/17 female) aged 14–41 years old, treated at our institution between 2015 and 2020 due to CSD or major deformity due to CLP (cleft lip and palate, uni- or bilateral) were enrolled in the study. The patients were assessed by analyzing the type of cleft, severity of the deformation, and the cleft width between the cleft edge point indexes according to Pradel et al. (2009) [17].

**Figure 1:**
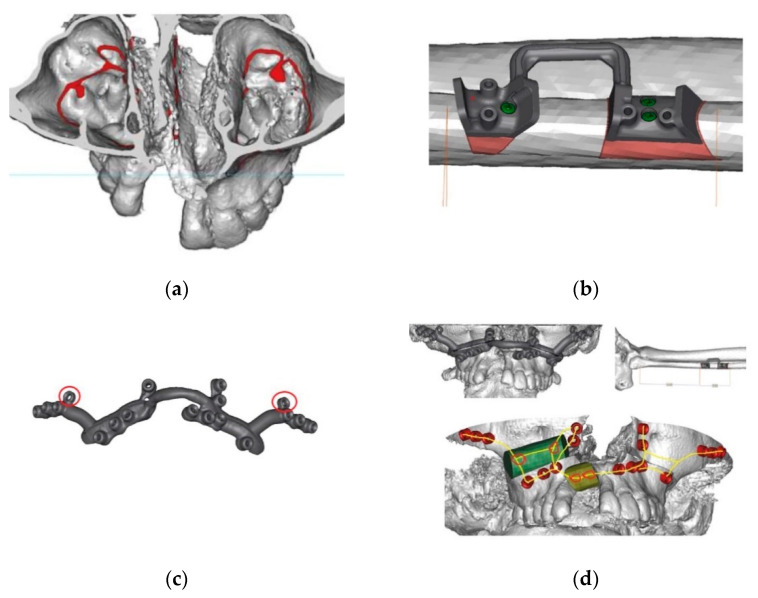
Examples of 3D-VSP planning and the design of custom guides and implants: (**a**) 3D reconstruction of the cleft right alveolus and maxilla, (**b**) guide for free fibula flap osteotomies, (**c**) customized fixation plate for bone graft fixation with simultaneous Le-Fort I osteotomy conceptualization and visualization. (**d**) Plan of the reconstructed maxilla with grafts and custom plates. (KLS Martin, Group, Tuttingen, Germany).

The protocol for cleft reconstruction assumed the following: preoperative examination and imaging (orthopantomogram, head and neck, abdomen, pelvis, and lower limb CT scans with contrast), virtual imaging and online planning with resective and reconstructive team, prosthodontist and laboratory team using KLS MARTIN IPS Case Designer (Figure 1), stereolithographic models, and production of 3D-VSP custom-made resection templates (KLS Martin, Group, Tuttingen, Germany). Type of the flap used for the reconstruction depended on cleft type, CSD size and shape, number of missing teeth, condition of pre-maxilla, necessity of maxilla and teeth extraction, soft tissue quality, and volume.

**Figure 2:**
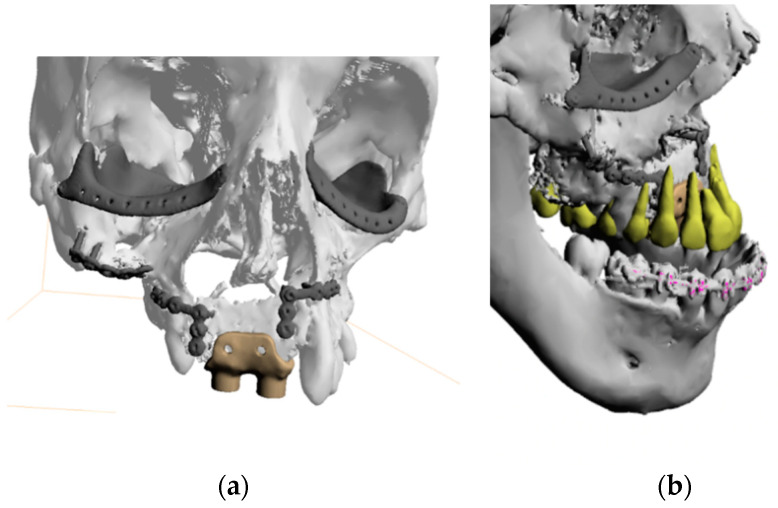
(**a**) 3D-VSP planning and the design of custom guides for dental implant placement. (**b**)Partial resorption of the graft resulted in insufficient bone volume for proper placement of dental implant that would provide physiological prosthetic reconstruction. 3D VSP provided information of re-augmentation necessity before final surgical correction of malloclusion by Le- Fort I advancement after dental implant placement (Patient 25, Table 1) (KLS Martin, Group, Tuttingen, Germany).

In the second stage of the reconstruction, individual guides for precise dental implant placement were designed and produced (Figure 2). The resection, microsurgical reconstructions and dental implant placement were performed by one team consisting of two maxillo-facial surgeons, one oncological surgeon, and prosthodontist.

## 3. Results

A total of 25 patients (8 male/17 female) aged 14–41 years old were enrolled in the study. All patients represented either a uni- or bilateral cleft lip and palate deformity (Table 1).

**Figure 3:**
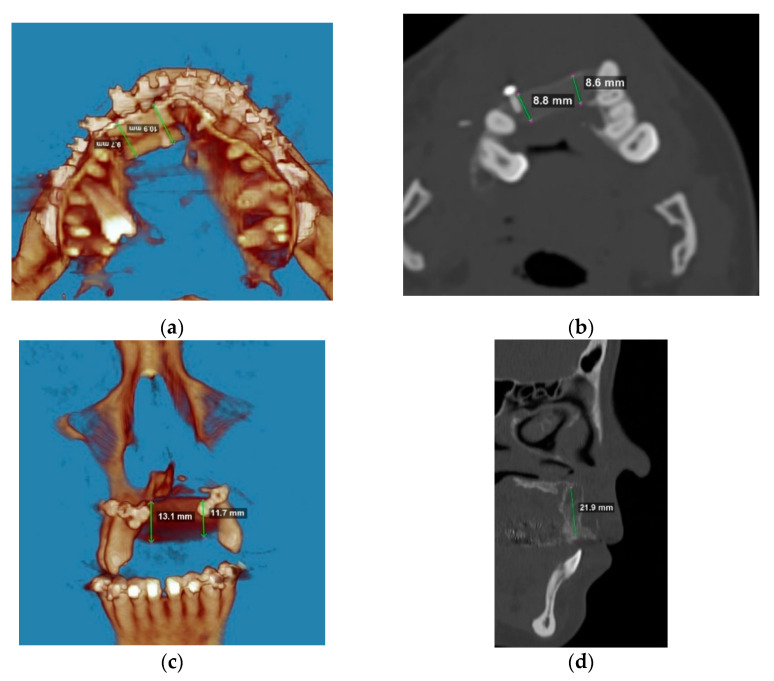
Representative photographs and 3D CT scans used for measurements of the graft: (**a**) 3D reconstruction in horizontal plane, (**b**) horizontal plane CT scan, (**c**) 3D reconstruction in coronal plane, (**d**)lateral plane CT scan.

The mean width of the cleft was 27.1 mm. All patients underwent 3D-assisted microvascular free flap reconstruction of the cleft. Iliac crest free flap (ICF) (n = 5), radial forearm flap (RFF) (n = 5), medial femoral condyle free flap (MFCFF) (n = 12), and fibula free flap (FFF) (n = 3) were used for reconstruction of the soft and hard tissues of the cleft maxilla, depending on the cleft width and deformity. Graft resorption was measured for up to 5 years after the surgery (Figure 3) (Table 2).

**Figure 4:**
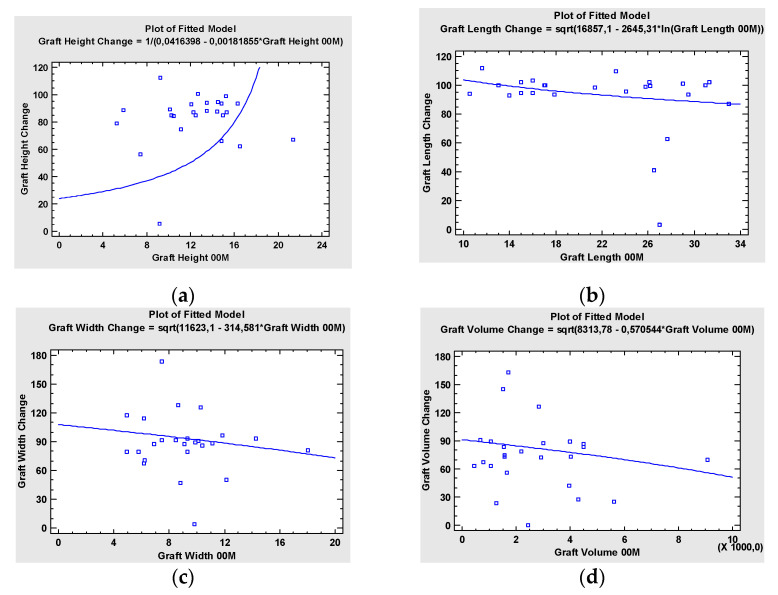
Simple regression Graft Height Change vs. Graft Height (**a**) showing insignificant levels of graft resorption in the horizontal dimension (height), *p* = 0.385; Simple regression Graft Length Change vs. Graft Length (**b**) showing insignificant levels of graft resorption in the sagittal dimension (length), *p* = 0.627 and Simple regression Graft Width Change vs. Graft Width (**b**) showing insignificant levels of graft resorption in the axial dimension (width), *p* = 0.862. Overall volume loss of the graft was insignificant at the follow ups: Simple Regression—Graft Volume Change vs. Graft Volume, *p* = 0.645. Figures (**c**,**d**) showing statistically insignificant changes of graft width and volume change with time.

During the follow ups, resorption of the graft and loss of volume were not statistically significant (*p* > 0.005) and (*p* = 0.645), respectively (Figure 4).

**Figure 5:**
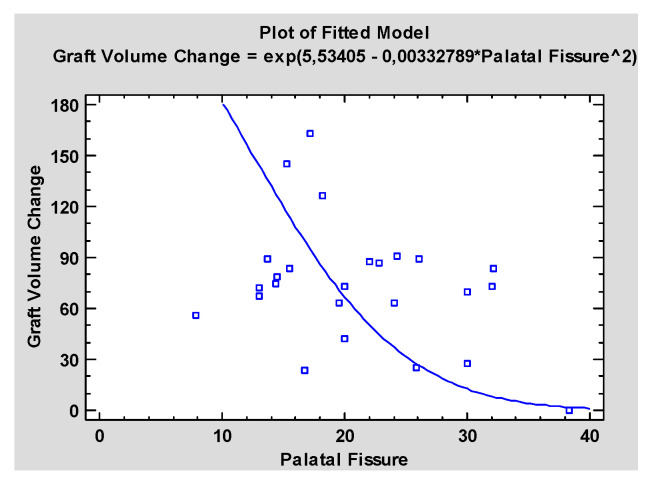
Simple Regression—Graft Volume Change vs. Palatal Fissure, indicating a moderately strong relationship between the variables.

There was a statistically significant correlation between width of the palatal fissure and the tendency of the graft for resorption. The wider the palatal fissure, the more significant the resorption of the graft in the follow up, *p* = 0.013 (Figure 5).

**Figure 6:**
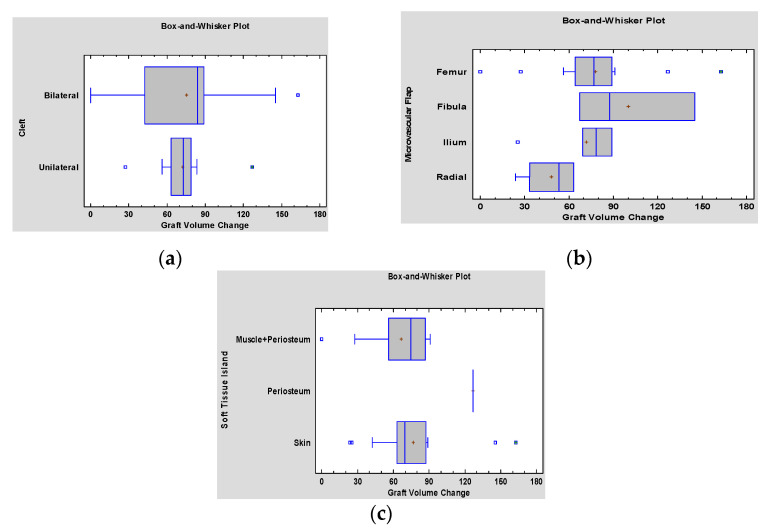
(**a**) Kruskal–Wallis Test for Graft Volume Change by Cleft analysis indicating insignificant correlation between type of cleft and graft bone volume loss (*p* = 0.5), (**b**) analysis of variances (ANOVA) showing that eventual bone resorption did not correlate with type of the flap used for the reconstruction (*p* = 0.299), (**c**) Kruskal–Wallis Test for Graft Volume Change by Cleft analysis, indicating insignificant correlation between the type of soft tissues island and graft bone volume loss (*p* = 0.378).

Bone resorption and volume loss of the graft did not correlate with the type of cleft (*p* = 0.5). (Figure 6a), type of flap (*p* = 0.299) (Figure 6b) nor soft tissue island (*p* = 0.378) (Figure 6c).

**Figure 7:**
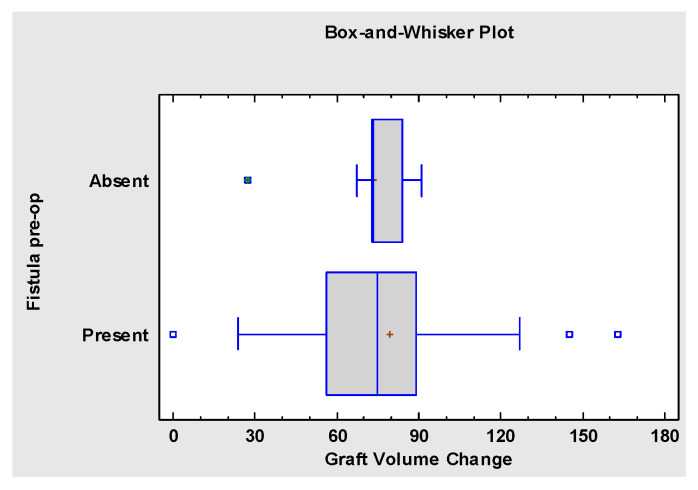
Kruskal–Wallis Test indicating insignificant correlation between presence of pre-operative oronasal fistula and severity of post-operative bone resorption.

The preoperative presence of the oronasal fistula did not affect the outcome of the graft healing and did not impact the post-operative graft bone volume loss (*p* = 0.867) (Figure 7).

**Figure 8:**
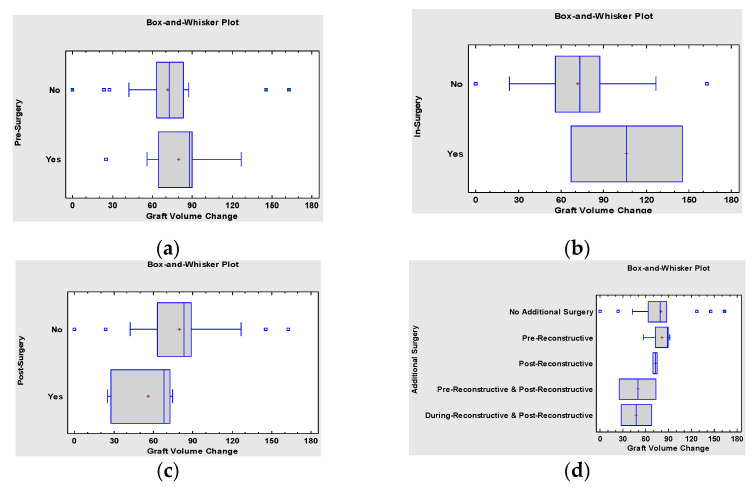
ANOVA Table for Graft Volume Change by: (**a**) Pre-Surgery indicating insignificant correlation between graft bone loss and presence of surgical procedures performed before final cleft reconstruction, e.g., LF1 osteotomy, LF1 osteotomy + TPD, BIMAX, Genioplasty (*p* = 0.6190), (**b**) In-surgery indicating insignificant correlation between graft bone loss and necessity of simultaneous operation, e.g., LF1 osteotomy, BIMAX (*p* = 0.1957) and (**c**) Post-surgery indicating insignificant correlation between graft bone loss and surgery performed after the reconstruction, e.g., additional bone graft, cleft rhinoplasty, LF or maxilla distraction (*p* = 0.1641), (**d**) Summary Statistics for Graft Volume Change ANOVA showing a between-group statistically insignificant (*p* = 0.6681).

The accompanying procedures during the cleft reconstruction (pre-, in- or post-reconstruction) did not have a statistically significant impact on post-operational graft bone and volume loss (Figure 8). A total of 8 patients required surgical preparation before reconstruction: Le-Fort 1 type maxillary osteotomy (LF-1) (n = 1), Le-Fort 1 type maxillary osteotomy and transverse palatal distraction (TPD) (n = 3), orthognathic surgery (BIMAX) (n = 2) and sliding genioplasty (n = 1). Simultaneous surgery was performed at the same time as the cleft reconstruction in two patients: BIMAX (n = 1), (LF-1) (n = 1). After the cleft reconstruction, additional surgery prior to dental implant placement was performed in 6 patients: bilateral sagittal split osteotomy (BSSO) of the mandible for correction of the occlusion (n = 1), free bone graft due to partial resorption of the microvascular bone graft (n = 2) and BIMAX (n = 1), and maxillary distraction osteogenesis (n = 2) for correction of the malocclusion 6 months after the reconstruction. None of the pre-, intra-, or postoperative additional surgeries affected microvascular bone graft resorption significantly, indicating that such an approach is predictable, safe and provides a sufficient base for dental implant treatment of the cleft maxilla (Figure 8).

**Figure 9:**
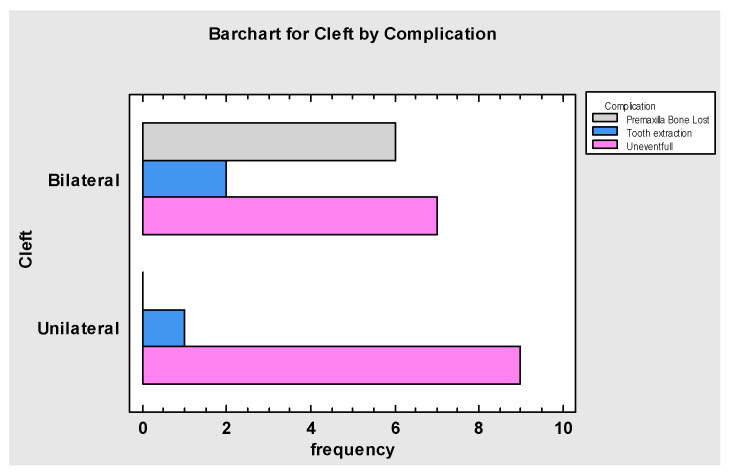
Tests of Independence showing that premaxilla resection was necessary in certain cases of bilateral CLP. Tooth extraction was more common in these patients (p = 0.0206). Unilateral CLP were mostly uneventful.

Severity of the cleft deformation, malocclusion and dental abnormalities were factors which contributed to the necessity to extract the teeth neighboring the cleft fissure in 3 patients at the time of the surgery (Figure 9). In 6 cases of bilateral CLP, premaxilla resection was necessary for stable graft implanting and fixing to provide sufficient reconstruction of the anterior maxilla nasal base.

**Figure 10:**
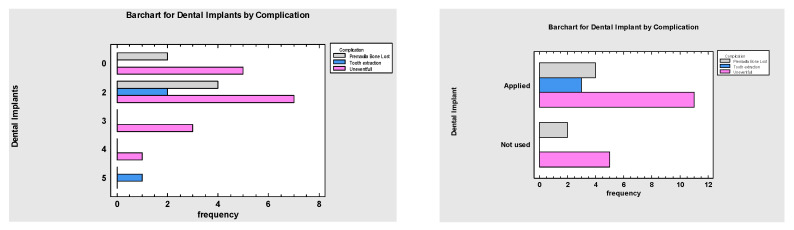
Frequency Table for Dental Implants by Complication, showing that those cases requiring tooth removal, premaxilla resection and partial bone loss of the graft received dental implant treatment, *p* = 0.005.

The most common complication was partial bone loss of the flap (n = 4), but only in 2 cases an additional bone graft was necessary prior to final dental implant treatment. There were two cases of flap failure due to thrombosis. Total loss of the graft was diagnosed, despite early intervention combined with thrombolysis and multiagent anticoagulation and antiplatelet therapy. In one patient, palatal fistula formation was diagnosed and treated by a locally advanced and rotated palatal flap. Among the 25 cohort, 18 patients underwent dental implant treatment (Figure 10). Each patient received 2–5 dental implants, depending on the CLP type and graft reconstruction. Two patients did not reach the point of dental implant treatment due to flap failure and were denied further treatment. Five patients did not receive dental implants due to ongoing orthodontic treatment after the microsurgical reconstruction at the time of the data gathering for this manuscript.

## 4. Discussion

**Figure 11:**
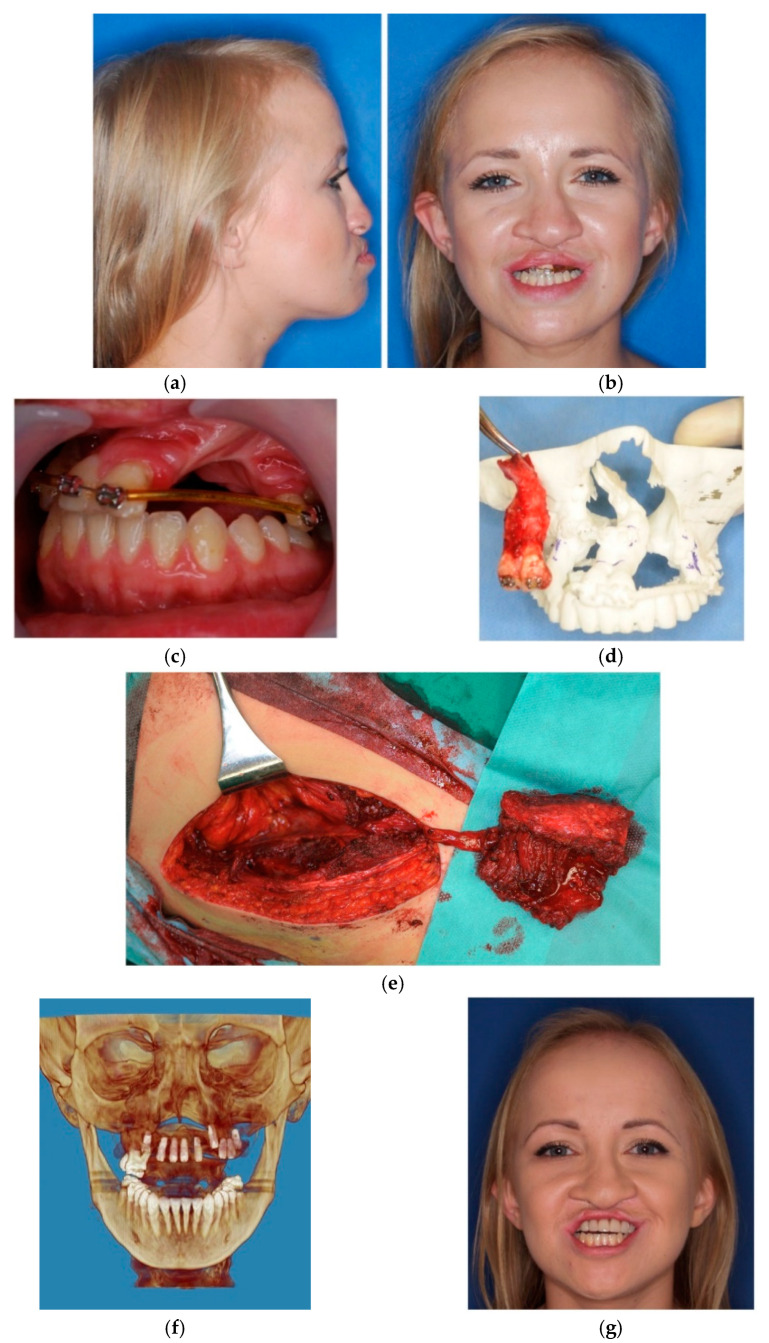
Photographs of patient treated due to bilateral cleft CSD and secondary acquired critical size deformation of the cleft maxilla: (**a**,**b**) pre-operative photographs, (**c**) cleft deformity and critical size defect requiring composite reconstruction, (**d**) intraoperative pre-maxilla resection, (**e**) prepared ICF flap on vessel peduncle prior harvesting and implantation, (**f**) dental implants placed 6 months after reconstruction, (**g**) final prosthetic reconstruction.

**Figure 12:**
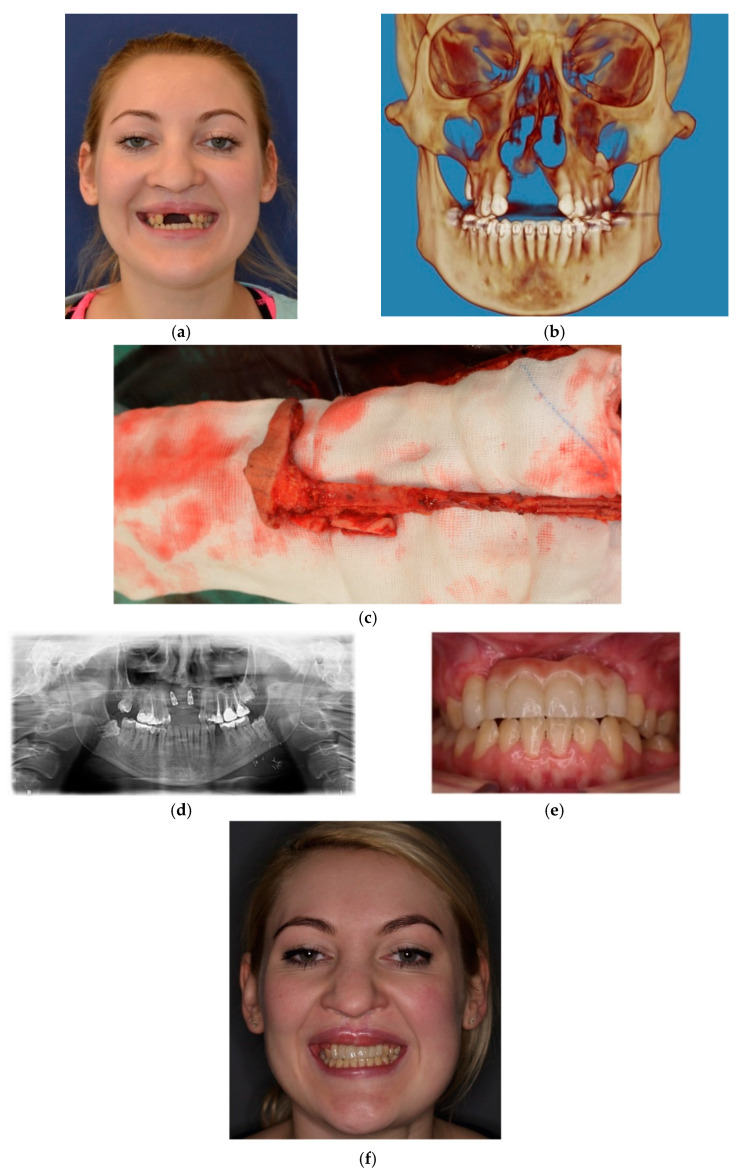
Photographs of a patient treated due to bilateral cleft CSD and secondary acquired huge deformation of the cleft maxilla and already lost premaxilla: (**a**) pre-operative photograph, (**b**) 3D reconstruction of the cleft in coronal plane, (**c**) intraoperative planning of RFF flap, (**d**) dental implants placed 6 months after reconstruction, (**e**) final prosthetic reconstruction and (**f**) facial appearance of the patient after full reconstruction of the cleft maxilla.

**Figure 13:**
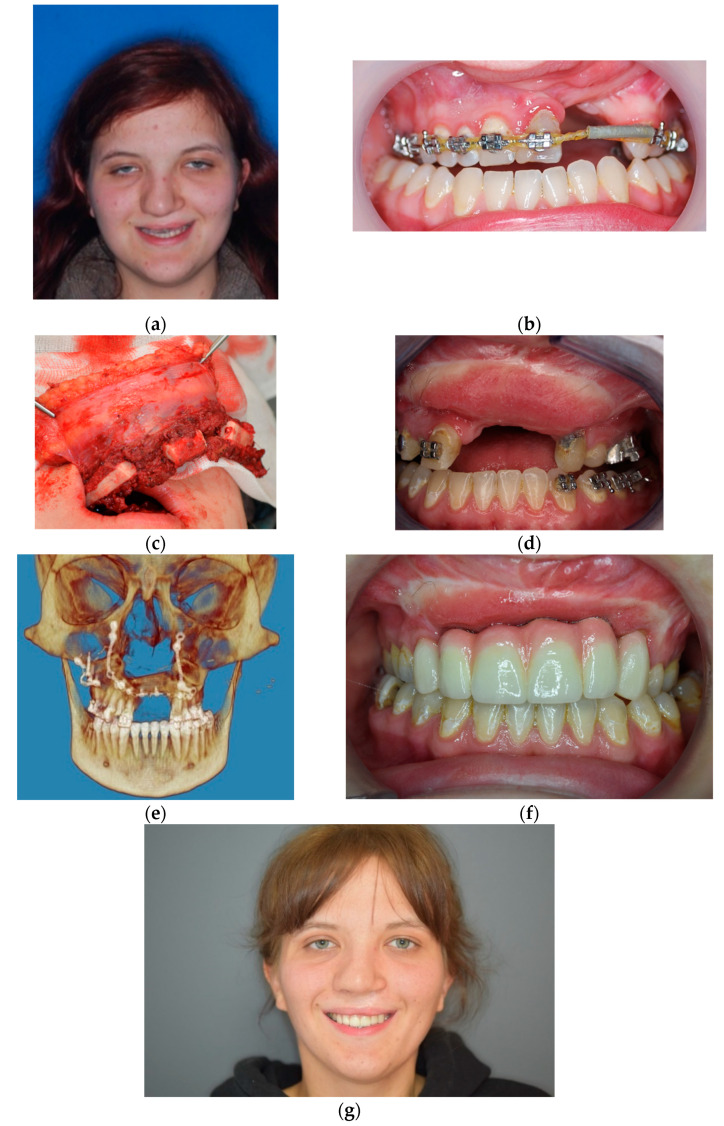
Photographs of a patient treated due to unilateral cleft CSD and secondary deformation of the cleft maxilla: (**a**) pre-operative photograph and (**b**) cleft CSD, (**c**) intraoperative placement of the FFF flap, (**d**) healed donor site, (**e**) 3D reconstruction of the operated CSD (**f**) final implant supported prosthetic superstructure, (**g**) facial appearance of the patient after full reconstruction of the cleft maxilla.

**Figure 14:**
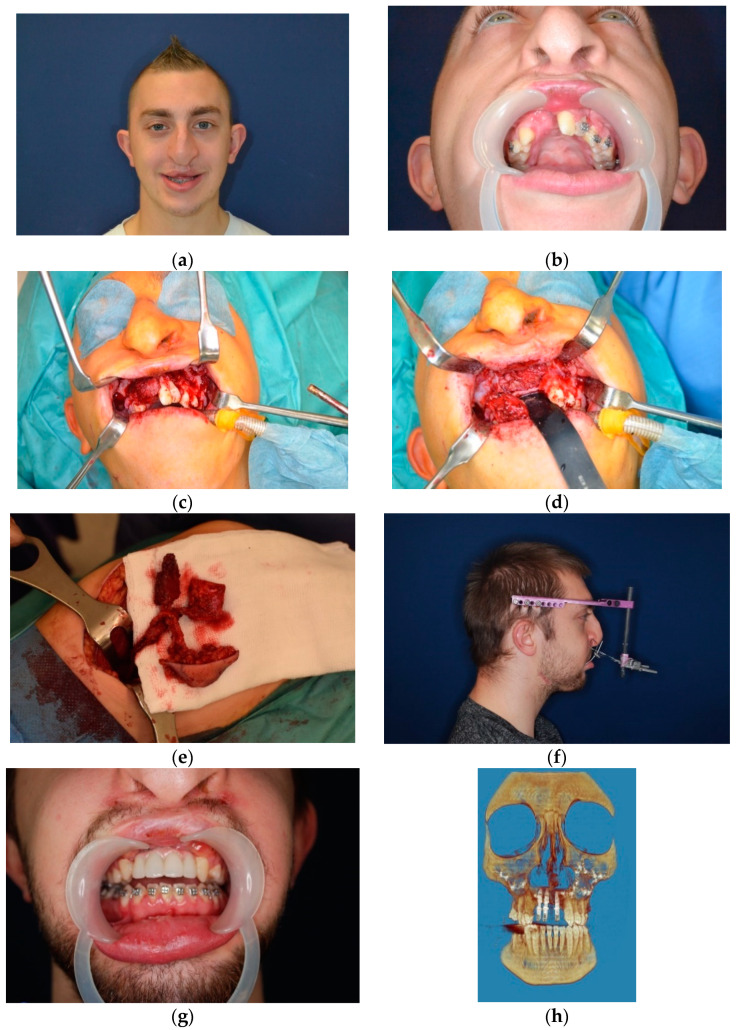
Photographs of a patient treated due to unilateral cleft CSD and secondary deformation of the cleft maxilla: (**a**) pre-operative photograph and (**b**) cleft CSD, (**c**) intraoperative preparation of the donor site and necessity of premaxilla resection due to bone deficiency around roots of the anterior teeth (**d**), (**e**) preparation of MFCFF flap, (**f**) Le Fort I osteotomy and anterior distraction of the graft with placed implants performed 6 months after first reconstruction, (**g**) final implant supported prosthetic superstructure, (**h**) 3D reconstruction after full reconstruction of the cleft maxilla.

The anterior maxilla is the most challenging anatomical area for oral rehabilitation with use of intraosseous dental implants [18]. Reconstructive treatment of the cleft maxilla is even more complicated, and the outcomes are frequently unsatisfactory. This is due to secondary deformations, malocclusion, bone loss induced by improper orthodontic treatment based on teeth and maxilla expansion leading to the formation of critical size defects and oro-nasal fistulas that cannot be reconstructed by simple grafting or augmentation procedures. The lack of bone support, scar tissue, inadequate amount of soft tissues and poor vascular sealing make dental implant treatment of the cleft maxilla almost impossible[19,20]. For critical size defects that also include soft tissue deficiencies and/or cases that require significant reconstruction of the jawbone, 3D-VSP supported microsurgical reconstruction is indicated. During the last 20 years, craniomaxillofacial surgery has begun to undertake reconstructive treatment of post ablative and posttraumatic defects as well as congenital or acquired deformations in adults, adolescents and children [21,22,23]. Prior to dental implant treatment, cleft maxilla requires reconstruction of both hard and soft tissues. Earlier techniques based on soft tissue transfer, while it provided sufficient closure of the oro-nasal fistula, failed to solve the problem of bone deficiency. In those cases, reconstruction required a second procedure based on free grafts or microvascular bone grafts placed into a previously prepared soft tissue bed [24,25]. Due to poor vascularity, it is common for free bone grafts to fail to heal sufficiently for the placing of dental implants. They are also characterized by severe levels of resorption, which prevents further implantation. Microvascular bone grafts are more predictable in this area, as they are supported by an artificially designed, separate blood vessel system. The use of a simultaneous bone–soft tissue flap offers significant benefits as it provides hard tissue support to stabilize the cleft maxilla and enables the placement of dental implants, where soft tissue islands form the future soft tissue seal around the implants and may also be used for simultaneous closure of fistula (if necessary). The type of flap and its design depend on the cleft type (uni- or bilateral), size of the defect, tissue deficiency, and presence/absence of palatal fistula at the time of the reconstruction (Table 3). In some cases of bilateral CLP with CSD, premaxilla is already lost prior to surgery or must be removed at the time of the final reconstruction in order to place fully functional dental implants [26,27,28]. Resection of pre-maxilla in that cases is more beneficial than preservation, as it provides full reconstruction of CSD with one graft and therefore, enables more predictable prosthetic reconstruction (Figure 11, Figure 12, Figure 13 and Figure 14).

The most commonly used and documented flap types for the reconstruction of cleft maxilla are radial forearm flap (RFF), medial femoral condyle free flap (MFCFF), iliac crest free flap (ICF), and fibula free flap (FFF).

RFF has a well-established proven track record of clinical applicability related to the reconstruction of orofacial defects. Batchelor and Palmer (1990) described RFF as the method of choice for the closure of palatal fistulas due to Wegener’s granulomatosis [29]. Chen et al. (1992) described RFF as an ideal flap for the reconstruction of CLP due to the thin, hairless skin and long vascular peduncle providing a functional and cosmetically pleasing reconstruction [30]. RFF has been clinically acceptable in the work of other authors. Barabas and Szabo (1993) used this type of reconstruction in the treatment of cleft palate [31]. McLeod et al. (1987) used RFF in the closure of palatal fistulas, reconstruction of anterior maxilla after premaxilla loss and alveolar cleft [32]. However, the major flaw in RFF is the necessity of skin grafting for the recipient site. The soft tissue part of the flap itself can in certain cases be too massive to be easily placed in the oral cavity. These disadvantages can be eliminated by the prelamination of the flap with oral mucosa. This technique assumes transplantation of the oral mucosa subcutaneously in the future recipient site. Fasico-mucosal flaps are thinner than fascio-cutanous flaps since no subcutaneous tissue is included, hence making the flap thinner, more elastic and with the potential for increased variety in configuration and positioning [33,34]. In order to increase the volume of the bony part of the RFF flap which is relatively thin, there are a number of possibilities related to bone splitting during the reconstruction.

For RFF there are incidental reports of radius fracture after graft harvesting. In this work we did not observe any incidents of recipient site fracture.

Iliac crest flap (ICF) is widely used in the reconstruction of orofacial defects, both as a free bone graft usually used for early alveolar grafting and, more recently, as a microvascular bone–soft tissue flap for the large reconstruction of jawbones, including mandible body ramus and TMJ due to the large amount of bone that can be obtained from the recipient site and the possibility of its intraoperative adaptation and modality [16]. However, in the reconstruction of cleft CSDs, the thick layer of muscle makes the use of ICF challenging in certain cases, and the iliac bone may represent a significant level of resorption [35,36]. Therefore, in this work we used the microvascular ICF flap in the reconstruction of large, bilateral clefts where significant amounts of bone and soft tissue material were necessary in order to reconstruct the whole anterior segment of the maxilla, where premaxilla was lost or required removal.

Medial femoral condyle free flap (MFCFF) was first described by Gaggl et al. (2008) and Kademani et al. (2009) in the reconstruction of the cleft maxilla. The advantages of this flap are associated with its anatomy, the possibility of adaptation and modality. The smaller size and periosteal flexibility make it ideal for conforming to alveolar maxillary defects, especially in situations where the size of the defect does not necessitate the use of larger flaps such as FFF or ICF [37,38]. Instead MFCFF was the most commonly used type for the reconstruction. Our observations are consistent with other researchers that this type of flap represents high bone quality with sufficient, yet thin, overlaying soft tissues that are of great value with regard to dental implant therapy and definitive maxillary reconstruction. The soft tissue thickness is around 2–3 mm, which enables easy implantation and subsequent super prosthetic reconstruction with a peri-implant tissue collar similar to the surrounding gingiva, which is not common in other types of flaps. The maximum size of the bone graft in MFCFF can be up to 70 mm, which is significantly less than in the case of FFF or ICF. In MFCFF there is no risk of bone fracture in the recipient site as in cases of RFF [38,39]. In our material, three patients treated with MFCFF required additive augmentation with bone grafts prior to final implant placement.

Fibula free flap (FFF) is commonly used in the reconstruction of the post ablative or congenital defects of the mandible as it provides graft lengths of up to 200 mm of the bone part and 160–210 mm of the soft tissue paddle [40,41]. It has a relatively thin layer of soft tissue, which may be beneficial with regard to dental implant placement but, due to the thick cortical bone and thin layer of cancellous bone, it is not susceptible to bending, modification or shape adaptation to the shape of the cleft CSD [35]. However, due to the rich blood supply and possibility of multiple osteotomies, FFF is feasible flap for sandwich techniques in the reconstruction of huge, composite tissue defects [42]. In our work, FFF was used in cases where simultaneous Le-Fort I osteotomies were performed for the ultimate correction of the jawbone relationship. The modified sandwich technique with FFF was applied for simultaneous reconstruction of the cleft maxilla and stabilization and augmentation of the LF-1 osteotomies and maxillary segments.

The major flaws of microvascular grafts are generally associated with their blood supply. Intraoperative adaptation of the shape and the desired size of the flap that fit the donor site frequently leads to injury of the supplying blood vessels, which causes flap ischemia and necrosis.

The formation of postoperative hematomas may lead to the same scenario. Early evacuation of the hematoma and flap revision are required in order to prevent flap necrosis due to thrombosis of the supplying blood vessels [43] or partial resorption due to impaired blood supply, which requires additional augmentation prior to dental implant placement [44]. The presented manuscript includes two cases of flap necrosis (one RFF and one FFF) and four cases requiring additive bone augmentation in the further stages of the oral rehabilitation—three cases of MFCFF and one of RFF.

In most cases, as the techniques and qualification of flaps for reconstructive surgeries have developed, in the case of patients with cleft palate, a skin island was abandoned for a muscle island to cover the flap, which achieved much better reconstruction of tissue similar to the mucous membrane of the attached gingiva, which can be observed in the results in the presented paper. The application of 3D planning and individual solutions for patients with a cleft palate is difficult and not particularly used. In those cases in which we wish to perform additional reconstructions, such as LF I, or rebuild a large bone void, we can use planning and individual molds and plates. In the case of RFF and MFCFF flaps, this is typically impossible. The use of molds for inserting implants is essential for very difficult bone conditions, despite the use of free flaps. The use of implant molds helps achieve an optimal and long-lasting aesthetic effect and improves the level of cooperation between the prosthetist and technician.

## 5. Conclusions

The application of microvascular tissue transfers is efficient and predictable method of the final reconstruction of the cleft maxilla with critical size defects. It provides sufficient bone volume for placement of dental implants and also supports maxilla stabilization. ICF flap is the most reliable in the reconstruction of bilateral, wide clefts with large CSD. MFCF flap is a method of choice in the reconstruction of unilateral clefts with partially preserved bone of the cleft fissure and secondary soft tissues deficiency. FFF is helpful in the reconstructions where subsequent LF-1 osteotomy, distraction, or orthognatic surgery is planned, as this graft serves as interposition, stabilizing element. Microvascular tissue transfers do not exhibit significant bone resorption what distinguishes them among contemporary reconstructive and bone augmentation procedures.

Within limitations of this report such as varied follow-up between the patients, use of 3D-VSP provides reliable and precise CSD reconstruction and final restoration of the maxilla with dental implants supporting the permanent prosthetic superstructures It also enables the performance of more complex procedures simultaneously with alveolar and maxillary grafting, such as Le-Fort 1 osteotomy and orthognathic surgery. However, larger cohort, longer follow-up, and comparison with other protocols, e.g., distraction osteogenesis are necessary in order to fully estimate longevity of such prosthetic reconstructions.

## Figures and Tables

**Table 1:** Patients treated due to cleft-associated critical size defect of the anterior maxilla.

Gender	Age [Year]	Diagnosis	Malignancy	Mandibulectomy Range	TMJ Replacement	Reconstruction Type	Follow-up [Years]	Additional Surgery	Final Treatment
Male	8	Fibroma Ossificans	Benign	Ramus only	Unilateral	*ICFF*	5	None	Ortodontic
Male	8	Fibrous Displasia	Benign	Ramus only	Unilateral	*ICFF*	5	None	Ortodontic
Female	10	Sarcoma	Cancer	Ramus & Body	Unilateral	*FFF*	4	None	Ortodontic & Dental Implants
Male	11	Sarcoma	Cancer	Ramus & Body	Unilateral	*FFF*	6	None	Ortodontic
Female	12	Severe Deformation	Benign	Ramus & Body	Bilateral	*FFF*	4	Ortognathic	Ortodontic
Male	13	Fibrous Displasia	Benign	Ramus & Body	Bilateral	*FFF*	2	None	Ortodontic
Male	13	Severe Deformation	Benign	Ramus & Body	Unilateral	*FFF*	5	Ortognathic	Ortodontic & Dental Implants
Male	13	Sarcoma	Cancer	Ramus & Body	Unilateral	*FFF*	5	None	Ortodontic
Male	13	Sarcoma	Cancer	Ramus & Body	Unilateral	*ICFF*	5	Reconstructive	Ortodontic & Dental Implants
Male	14	Amelobastoma	Benign	Ramus & Body	Unilateral	*FFF*	4	None	Ortodontic & Dental Implants
Male	15	Fibrous Displasia	Benign	Ramus & Body	Unilateral	*FFF*	2	None	Ortodontic
Female	15	Amelobastoma	Benign	Ramus & Body	Unilateral	*FFF*	5	Reconstructive	Ortodontic & Dental Implants
Female	17	Amelobastoma	Benign	Ramus & Body	Unilateral	*ICFF*	5	Ortognathic	Ortodontic & Dental Implants
Male	14	Central Cell Giant Granuloma	Benign	Ramus & Body	Unilateral	*FFF*	4	None	Ortodontic & Dental Implants

**Table 2:** Graft measurements at the day of reconstruction and follow up (12–60 months).

Gender	Age [Years]	Mandibulectomy Range	TMJ Replacement	Reconstruction Type	MIOpre [mm]	MIOpost [mm]	SNBpre [deg.]	SNBpost [deg.]	Ramus OP [mm]	Ramus OP’ [mm]	Body OP [mm]	Body OP’ [mm]	Ramus C [mm]	Ramus C’ [mm]	Body C [mm]	Body C’ [mm]	Assymetry [mm]
Male	8	Ramus only	Unilateral	f_Iliac_F	5	30	69.2	72.5	19.3	20.7	47.3	50	52	54	47.2	51	6
Male	8	Ramus only	Unilateral	f_Iliac_F	10	35	77	74	28.7	28.7	68.4	68.4	49	51	56	57	0
Female	10	Ramus & Body	Unilateral	f_Fibula_F	0.5	30	77.2	74.7	41	43.3	89	91	58	65	68	70	0
Male	11	Ramus & Body	Unilateral	f_Fibula_F	5	35	78.5	79	43	43.5	63.8	64	50	53	53	56	0
Female	12	Ramus & Body	Bilateral	f_Fibula_F	25	35	56	82.6	n/a	n/a	n/a	n/a	n/a	n/a	n/a	n/a	0
Male	13	Ramus & Body	Bilateral	f_Fibula_F	5	35	74	80	n/a	n/a	n/a	n/a	n/a	n/a	n/a	n/a	3
Male	13	Ramus & Body	Unilateral	f_Fibula_F	0.5	35	69.1	75.5	45.8	51.8	50.5	58.6	52.7	53	54.8	61	5
Male	13	Ramus & Body	Unilateral	f_Fibula_F	15	35	77.2	75.4	29.9	31	35.4	37.5	56	57.5	70	77.4	5
Male	13	Ramus & Body	Unilateral	f_Iliac_F	40	40	65	73.1	34	42.2	41	42.9	51	56	67	70	0
Male	14	Ramus & Body	Unilateral	f_Fibula_F	10	40	64	76	37	39	98	99	47.7	56	62.1	72	4
Male	15	Ramus & Body	Unilateral	f_Fibula_F	10	35	72	74.4	41.6	43	27.5	27.5	45	56	49	72	6
Female	15	Ramus & Body	Unilateral	f_Fibula_F	10	45	69.4	71	32.3	37.2	56.3	61.5	53	61	64	73	7
Female	17	Ramus & Body	Unilateral	f_Iliac_F	35	45	68	74	30.3	30.5	30	29	48	58	49	61	0
Male	14	Ramus & Body	Unilateral	f_Fibula_F	10	40	71	74	35	38.6	31.1	47.2	55	59	60	67	4

**Table 3:** Comparison of flaps used in the microsurgical reconstruction of the cleft maxilla with regard to dental implant therapy.

Type of Flap	Advantages	Disadvantages	Indications
Radial forearm flap (RFF)	- long supporting vessel peduncle - thin, hairless skin pedicle - cortico—cancellous bone of good quality for dental implants	- risk of radius fracture - non-aesthetic scarring in the donor site - limited amount of bone- for 2–3 dental implants - diameters of the supplying vessels are greater than the facial vessels - skin is poorly epithelialized and not beneficial for prosthetic reconstruction	- bone dehiscence of satisfying soft tissue conditions requiring reconstruction up to 3 dental implants
Medial femoral condyle free flap (MFCFF)	- high modality - comfortable adaptation to recipient’s site shape and size - bone and soft tissue of good quality enabling aesthetic implant supported reconstructions	- maximum graft size of 30–70 mm - short vessel pedicle - mono-cortical graft - proper for defects with bone present at distal side of the CSD	- bone dehiscence with preserved palatal wall requiring reconstruction up to 3 dental implants along with soft tissue augmentation and volumization.
Iliac crest free flap (ICF)	- optimal for large reconstructions - adaptation and modality	- moderate resorption over time - thick muscle layer which may require secondary contouring - short vessel pedicle	- large reconstructions of the maxilla where 3–5 and more dental implants are required without necessity of flap splitting and need for augmentation and volumization of the soft tissues.
Fibula free flap (FFF)	- long bone graft up to 200 mm - thin soft tissue lining enabling aesthetic implant reconstruction - ease of flap design and harvest - multiple osteotomies possible due to rich blood supply	- stiffness making the graft non-modal to cleft CSDs of the maxilla in certain cases	- medium and medium- large bone defects requiring placement of more than 3 implants, interposition and stabilization of the maxillary bones in the anterior area and layer closure of soft and hard tissues of the cleft, also used in simultaneous maxillary advancement to reconstruct osteotomies

## References

[1] Agrawal K. (2009). Cleft palate repair and variations. Indian J. Plast. Surg..

[2] Cassi D., Di Blasio A., Gandolfinini M., Magnifico M., Pellegrino F., Piancino M.G. (2017). Dentoalveolar Effects of Early Orthodontic Treatment in Patients With Cleft Lip and Palate. J. Craniofacial Surg..

[3] Coots B.K. (2012). Alveolar Bone Grafting: Past, Present, and New Horizons. Semin. Plast. Surg..

[4] Mahajan R., Ghildiyal H., Khasgiwala A., Muthukrishnan G., Kahlon S. (2017). Evaluation of Secondary and Late Secondary Alveolar Bone Grafting on 66 Unilateral Cleft Lip and Palate Patients. Plast. Surg..

[5] Duskova M., Kotova M., Sedlackova K., Leamerova E., Horak J. (2007). Bone Reconstruction of the Maxillary Alveolus for Subsequent Insertion of a Dental Implant in Patients with Cleft Lip and Palate. J. Craniofacial Surg..

[6] Sandor G., Carmichael R.P., Brkovic B.M. (2010). Dental implants placed into alveolar clefts reconstructed with tongue flaps and bone grafts. Oral Surg. Oral Med. Oral Pathol. Oral Radiol. Endodontol..

[7] Takahashi T., Inai T., Kochi S., Fukuda M., Yamaguchi T., Matsui K., Echigo S., Watanabe M. (2008). Long-term follow-up of dental implants placed in a grafted alveolar cleft: Evaluation of alveolar bone height. Oral Surg. Oral Med. Oral Pathol. Oral Radiol. Endodontol..

[8] Nawfal F., Hicham B., Achraf B., Rachid B., F N., B H. (2014). Repair of large palatal fistula using tongue flap. Afr. J. Paediatr. Surg..

[9] Shash H., Al-Halabi B., Jozaghi Y., Aldekhayel S., Gilardino M.S. (2016). A Review of Tissue Expansion-Assisted Techniques of Cleft Palate Repair. J. Craniofacial Surg..

[10] Van Damme P.A., Freihofer H.P.M. (1996). Palatal Mucoperiosteal Expansion as an Adjunct to Palatal Fistula Repair: Case Report and Review of the Literature. Cleft Palate-Craniofacial J..

[11] Rachmiel A. (2007). Treatment of Maxillary Cleft Palate: Distraction Osteogenesis Versus Orthognathic Surgery—Part One: Maxillary Distraction. J. Oral Maxillofac. Surg..

[12] Cheung L., Chua H. (2006). A meta-analysis of cleft maxillary osteotomy and distraction osteogenesis. Int. J. Oral Maxillofac. Surg..

[13] Zemann W., Kruse A.L., Lüebbers H.T., Jacobsen C., Metzler P., Obwegeser J.A. (2011). Microvascular Tissue Transfer in Cleft Palate Patients. J. Craniofacial Surg..

[14] Liou E.J.W., Chen P.K.T., Huang C.S., Chen Y.R. (2000). Interdental Distraction Osteogenesis and Rapid Orthodontic Tooth Movement: A Novel Approach to Approximate a Wide Alveolar Cleft or Bony Defect. Plast. Reconstr. Surg..

[15] Dolanmaz D., Karaman A.I., Durmus E., Malkoc S. (2003). Management of alveolar clefts using dento-osseous transport distraction osteogenesis. Angle Orthod..

[16] Dowgierd K., Pokrowiecki R., Borowiec M., Kozakiewicz M., Smyczek D., Krakowczyk Ł. (2021). A Protocol for the Use of a Combined Microvascular Free Flap with Custom-Made 3D-Printed Total Temporomandibular Joint (TMJ) Prosthesis for Mandible Reconstruction in Children. Appl. Sci..

[17] Pradel W., Senf D., Mai R., Ludicke G., Eckelt U., Lauer G. (2009). One-stage palate repair improves speech outcome and early maxillary growth in patients with cleft lip and palate. J. Physiol. Pharmacol..

[18] Belser U.C., Schmid B., Higginbottom F., Buser D. (2004). Outcome analysis of implant restorations located in the anterior maxilla: A review of the recent literature. Int. J. Oral Maxillofac. Implant..

[19] Borba A.M., Borges A.H., da Silva C.S.V., Brozoski M.A., Naclério-Homem M.D.G., Miloro M. (2014). Predictors of complication for alveolar cleft bone graft. Br. J. Oral Maxillofac. Surg..

[20] Alexandra M.P.E., Andre B., Priscila L.C., Patricia N.T. (2017). Alveolar Bone Graft: Clinical Profile and Risk Factors for Complications in Oral Cleft Patients. Cleft Palate-Craniofacial J..

[21] Dowgierd K., Krakowczyk Ł. (2019). The use of microsurgical reconstruction in treatment of craniofacial defects in paediatric patients. Int. J. Oral Maxillofac. Surg..

[22] Shahzad F. (2020). Pediatric Mandible Reconstruction: Controversies and Considerations. Plast. Reconstr. Surg. Glob. Open.

[23] Fisher J., Jackson I.T. (1989). Microvascular surgery as an adjunct to craniomaxillofacial reconstruction. Br. J. Plast. Surg..

[24] Futran N.D. (2001). Retrospective case series of primary and secondary microvascular free tissue transfer reconstruction of midfacial defects. J. Prosthet. Dent..

[25] Ninkovic M., Hubli E.H., Schwabegger A., Anderl H. (1997). Free Flap Closure of Recurrent Palatal Fistula in the Cleft Lip and Palate Patient. J. Craniofacial Surg..

[26] Monasterio F.O., Santamaría E., Morales D., Morales C., Yudovich M., Ramos F.S. (2009). Reconstruction of the Premaxilla. J. Craniofacial Surg..

[27] Holmes J.D., Aponte-Wesson R. (2010). Dental Implants After Reconstruction with Free Tissue Transfer. Oral Maxillofac. Surg. Clin. N. Am..

[28] Urken M.L., Buchbinder D., Costantino P.D., Sinha U., Okay D., Lawson W., Biller H.F. (1998). Oromandibular Reconstruction Using Microvascular Composite Flaps. Arch. Otolaryngol. Head Neck Surg..

[29] Batchelor A., Palmer J. (1990). A novel method of closing a palatal fistula: The free fascial flap. Br. J. Plast. Surg..

[30] Chen H.-C., Ganos D.L., Coessens B.C., Kyutoku S., Noordhoff M.S. (1992). Free Forearm Flap for Closure of Difficult Oronasal Fistulas in Cleft Palate Patients. Plast. Reconstr. Surg..

[31] Barabás J., Szabó G. (1993). Closure of cleft palate in adult patients, using a forearm flap with microvascular radial artery II. Fogorvosi Szle..

[32] MacLeod A., Morrison W., McCann J., Thistlethwaite S., VanderKolk C., Ryan A. (1987). The free radial forearm flap with and without bone for closure of large palatal fistulae. Br. J. Plast. Surg..

[33] Millesi W., Rath T., Millesi-Schobel G., Glaser C. (1998). Reconstruction of the floor of the mouth with a fascial radial forearm flap, prelaminated with autologous mucosa. Int. J. Oral Maxillofac. Surg..

[34] Kim G.G., Halvorson E.G., Hang A.X., Pederson W.C., De Santis G., Hackman T.G. (2012). Prelamination of Radial Forearm Free Flap with Buccal Mucosa. Otolaryngol. Neck Surg..

[35] Kelly C.P., Moreira-Gonzalez A., Ali M.A., Topf J., Persiani R.J., Jackson I.T. (2004). Vascular Iliac Crest With Inner Table of the Ilium as an Option in Maxillary Reconstruction. J. Craniofacial Surg..

[36] Mücke T., Hölzle F., Loeffelbein D.J., Ljubic A., Kesting M., Wolff K.-D., Mitchell D.A. (2011). Maxillary reconstruction using microvascular free flaps. Oral Surgery Oral Med. Oral Pathol. Oral Radiol. Endodontology.

[37] Kademani D., Salinas T., Moran S.L. (2009). Medial Femoral Periosteal Microvascular Free Flap: A New Method for Maxillary Reconstruction. J. Oral Maxillofac. Surg..

[38] Gaggl A., Bürger H., Chiari F.M. (2008). The Microvascular Osteocutaneous Femur Transplant for Covering Combined Alveolar Ridge and Floor of the Mouth Defects: Preliminary Report. J. Reconstr. Microsurg..

[39] Werle A.H., Tsue T.T., Toby E.B., Girod D.A. (2000). Osteocutaneous radial forearm free flap: Its use without significant donor site morbidity. Otolaryngol. Neck Surg..

[40] Landes C., Korzinskas T., Dehner J.-F., Santo G., Ghanaati S., Sader R. (2014). One-stage microvascular mandible reconstruction and alloplastic TMJ prosthesis. J. Cranio-Maxillofacial Surg..

[41] Ni Y., Lu P., Yang Z., Wang W., Dai W., Qi Z.-Z., Duan W., Xu Z.-F., Sun C.-F., Liu F. (2018). The application of fibular free flap with flexor hallucis longus in maxilla or mandible extensive defect: A comparison study with conventional flap. World J. Surg. Oncol..

[42] Weitz J.S., Kreutzer K., Bauer F., Wolff K.-D., Nobis C., Kesting M.R. (2015). Sandwich flaps as a feasible solution for the management of huge mandibular composite tissue defects. J. Cranio-Maxillofacial Surg..

[43] Schmelzeisen R., Schliephake H. (1998). Interdisciplinary microvascular reconstruction of maxillary, midfacial and skull base defects. J. Cranio-Maxillofacial Surg..

[44] Turk A.E., Chang J., Soroudi E.A., Hui K., Lineaweaver W.C. (2000). Free Flap Closure in Complex Congenital and Acquired Defects of the Palate. Ann. Plast. Surg..

